# Risk Factors For Progression From Biochemical Leak to Clinically Relevant Postoperative Pancreatic Fistula After Pancreaticoduodenectomy. The Key of the Lock: Prognostic Nutritional Index

**DOI:** 10.5152/tjg.2024.24425

**Published:** 2024-11-04

**Authors:** Mehmet Can Aydin, Oguzhan Ozsay, Kagan Karabulut, Recep Bircan, Fatih Atalay, Mehmet Batuhan Ors

**Affiliations:** 1 Department of Gastrointestinal Surgery, Bursa State Hospital, Bursa, Türkiye; 2 Department of General Surgery, Ondokuz Mayıs University, Samsun, Türkiye; 3 Department of General Surgery, Çarşamba State Hospital, Samsun, Türkiye

**Keywords:** Prognostic nutritional index, postoperative pancreatic fistula, biochemical leak

## Abstract

**Background/Aims:**

Postoperative pancreatic fistula (POPF), which is considered the most frightening complication after pancreaticoduodenectomy (PD), continues to be a serious problem even in experienced centers. In the present study, we aimed to determine the risk factors that increase the progression from biochemical leak (BL) to clinically relevant postoperative pancreatic fistula (CR-POPF) after PD.

**Materials and Methods:**

We retrospectively analyzed the data of 152 patients who underwent PD. A total of 71 patients who developed POPF were included in the study and divided into two groups: 52 patients remained in the BL stage and 19 patients progressed from BL to CR-POPF. The groups were compared in terms of preoperative data, perioperative findings, and postoperative results. Risk factors for progression from BL to CR-POPF were analyzed.

**Results:**

Preoperative prognostic nutritional index (PNI) was significantly lower in the CR-POPF group compared to the BL group (35.6 (30.1-47.9) vs 41.6 (33.5-58), *P* < .001). Receiver operating characteristic (ROC) curve analysis showed that the best cutoff of preoperative PNI value for predicting this progression was 38 (AUC = 0.835; 95% CI, 0.717-0.953; *P* = .001). While the progression rate was 58.3% in the group with PNI < 38, it was 10.6% with PNI ≥ 38. In univariate and multivariate analysis, preoperative PNI value was the only independent risk factor for progression from BL to CR-POPF after PD (OR, 15.428; 95% CI, 3.714-64.085; *P* < .01).

**Conclusion:**

Preoperative PNI value is an important parameter predicting the progression from BL to CR-POPF after PD.

**Main Points**

- The preoperative Prognostic Nutritional Index (PNI) is an important parameter predicting the progression from Biochemical Leak (BL) to Clinically Relevant Postoperative Pancreatic Fistula (CR-POPF) after pancreaticoduodenectomy (PD).
- More attention should be given to the possibility of patients having BL and low preoperative PNI value progressing to CR-POPF after PD.
- The patients with a preoperative PNI value < 38 and who have BL may benefit from earlier diagnostic imaging, interventions, somatostatin analogs, longer drainage time, or nutritional support.

## Introduction

Pancreaticoduodenectomy (PD) has remained the only curative treatment option for periampullary tumors since it was first described in 1935.^1^ Although the initial high mortality rate decreased to less than 5% over time, the morbidity rate still continues to be around 40%-50%, even in experienced centers.^2^ The most important cause of the morbidity, and the biggest concern of pancreatic surgeons, is postoperative pancreatic fistula (POPF). The main reason for this concern is the complications that may develop due to POPF, such as intra-abdominal bleeding, abscess, and sepsis, which may cause mortality, prolonged hospital stay, and high healthcare costs.^3^

Postoperative pancreatic fistula was defined and categorized as Grade A, B, and C by the International Study Group of Pancreatic Surgeons (ISGPS) in 2005 so that a common language could be used by all pancreatic surgeons, and this definition was updated in 2016.^4^ The most striking change in the update was that the condition formerly defined as “Grade A fistula” should no longer be considered a true fistula as it had no clinical significance and was renamed as “Biochemical Leak” (BL). In subsequent studies, while only patients with Grades B and C fistulas were considered to have POPF, BL was grouped with patients who did not develop any fistulas according to ISPGS definition.^5,6^ However, the progression from BL, which was defined as the initial stage of POPF diagnosis by the ISGPS, to Clinically Relevant Postoperative Pancreatic Fistula (CR-POPF), has been almost never addressed in the literature, and the risk factors for this progression have not been examined.

In the present study, we aimed to investigate the risk factors for progression from BL to CR-POPF after PD. To the best of our knowledge, this is the first study conducted from this perspective regarding this deficiency in the literature. 

## Materials and Methods

This study was approved by the local ethical committee of Ondokuz Mayıs University School of Medicine (approval no: 2022/579, date: December 22, 2022). The data of the 152 patients who underwent PD from December 2022 to April 2024 were examined. Prospectively collected data from the patients were retrospectively analyzed. The decision for surgery was made by the multidisciplinary council. The written informed consent was obtained from all of the patients preoperatively. The operations were performed by three senior surgeons. The details of the surgical procedures and perioperative management have been reported in our previous studies.^7,8^ The patients who underwent emergency surgery and had non-tumor diagnoses were excluded at first. Secondly, patients with no POPF (BL or CR-POPF) were excluded. Finally, 71 patients were included in the study. The patients were divided into two groups: those who remained in BL stage (n = 52) and those who progressed to CR-POPF (n = 19). The patients’ preoperative demographics, body mass index, previous medical and previous upper abdominal surgical history, American Society of Anesthesiologists score, preoperative Prognostic Nutritional Index (PNI) value, preoperative biliary drainage status, tumor location, perioperative blood transfusion, operation time, pancreatic texture, diameter of Wirsung, C-reactive protein (the highest value between the 1 and 3 days postoperatively), postoperative complications, re-operation, length of hospital stay, mortality, and definitive pathological results were analyzed. The PNI was calculated as 10× serum albumin (g/dL) + 0.005 × total lymphocyte count (per mm^3^)^9^ and venous blood samples were drawn within three days before surgery. Preoperative biliary drainage was performed via endoscopic or percutaneous methods. The diameter of Wirsung was categorized as ≥3 mm and <3 mm, and the pancreatic texture was recorded as soft or hard, according to the surgeons’ definition, perioperatively. The modified Blumgart technique was performed for all pancreaticojejunostomy anastomoses, as we reported previously.^7^ Enteral and parenteral nutritional support was not given to any of the patients in the preoperative period or until the time of CR-POPF diagnosis postoperatively. Somatostatin analogs were not used, except for some patients who had CR-POPF postoperatively. Postoperative complications were graded according to the Clavien–Dindo classification, and ≥3 were accepted as severe complications.^10^ The diagnosis of BL or CR-POPF was made according to the ISGPS 2016 update.^4^ According to the ISGPS, if the amylase level of the drain fluid was 3 times higher than our institutional upper limit on the 3rd postoperative day and there was not any clinically relevant change, it was graded as BL. When a clinically relevant condition was added to this stage, such as signs of infection without organ failure, the need for percutaneous or endoscopic drainage, angiographic procedures for bleeding, or persistent drainage for more than 3 weeks, it was defined as Grade B POPF. If a Grade B POPF leads to organ failure, clinical instability such that a re-operation is needed, or mortality, it was defined as Grade C POPF. Delayed gastric emptying was defined as an inability to take a standard oral diet or the need for nasogastric decompression beyond the postoperative day 7, and it was graded according to the ISPGS.^11^ Mortality was defined as death within 90 days, postoperatively.

### Statistical Analysis

Patients were divided into two cohorts based on the existence of BL or CR-POPF, and all data were compared. Continuous variables presented as median (minimum-maximum) or mean (±SD) were compared using the T-test or Mann–Whitney *U* test. Categorical variables were reported as numbers with percentages and compared using the Fisher exact test. The optimum PNI threshold value associated with progression from BL to CR-POPF was defined by receiver operating characteristic curve (ROC) analysis and expressed as the area under the curve (AUC). In addition, sensitivity, specificity, positive predictive value (PPV), and negative predictive value (NPV) were calculated, and the discrimination threshold was adjusted for easy clinical utilization. Univariate and multivariate logistic regression analyses (with the enter method) were performed to discover independent factors associated with the progression from BL to CR-POPF. In order to enter multivariable analysis, factors had to be statistically significant in univariate analysis (*P* < .25). Results were presented as odds ratios (ORs) with 95% confidence intervals (CIs). *P*-value <.05 was considered to be statistically significant. All statistical analyses were performed using IBM SPSS Statistics for Mac, Version 29.02 (IBM Corp., Armonk, New York, USA).

## Results

**Figure 1.**
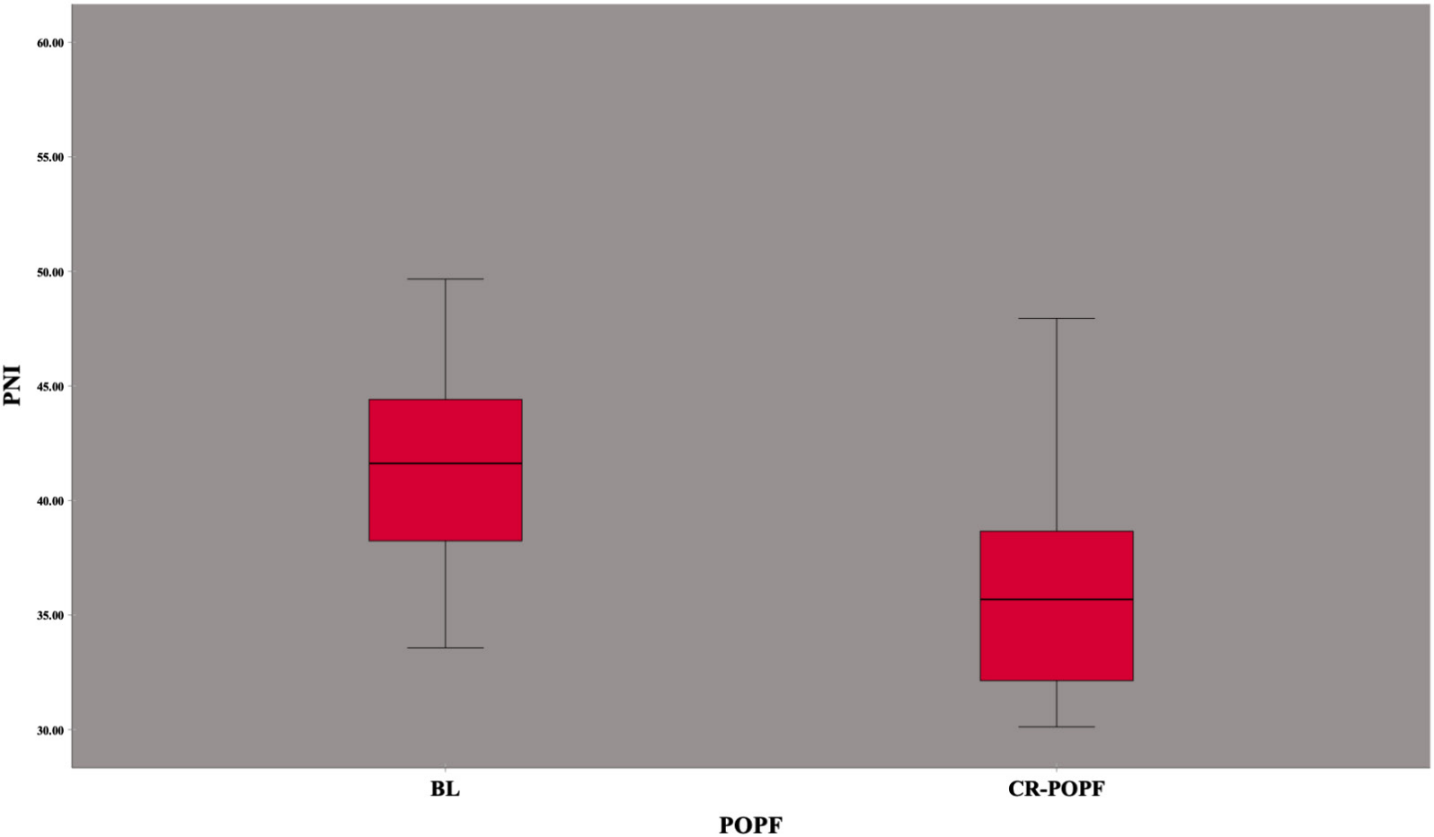
Distribution of preoperative PNI in patients with BL and CR-POPF.

The mean age of the whole study group was 63 ± 11 years, and 30 (42.3%) of them were female. The demographics, preoperative findings, and definitive pathological results are summarized in Table 1. There was no significant difference between the groups except for the preoperative PNI value, which was significantly higher in the BL group (41.6 (33.5-58) vs 35.6 (30.1-47.9), *P* < .001) (Figure 1). 

The perioperative findings and postoperative results are summarized in Table 2. Perioperative findings were similar between groups. When postoperative results were evaluated, as expected, serious complications (57.9% vs. 15.8%, *P* < .001), CR-POPF-related re-operation (21% vs. 0%, *P* = .025), and mortality (15.8% vs. 0%, *P* = 0.017) rates were higher, and length of hospital stay (21 (8-57) vs. 13 (6-30), *P* < .001) was longer, in the CR-POPF group. 

In all of the 71 patients included in the study, the amylase value of the drain fluid taken on the 3rd postoperative day was 3 times above the upper limit of our institution (100 IU/L). Nineteen (26.7%) of these patients progressed from BL to CR-POPF, and the median progression time was 9 (5-19) days. While 15 of them (79%) remained in Grade B stage, 4 (21%) patients progressed to Grade C. Patients with Grade B POPF were treated with percutaneous drainage (n = 3, 20%) or persistent drainage for more than 3 weeks (n = 12, 80%). All of the patients with Grade C POPF were re-operated, 2 due to massive intra-abdominal hemorrhage and 2 due to intra-abdominal sepsis, and mortality was observed in 3 of them.

**Figure 2.**
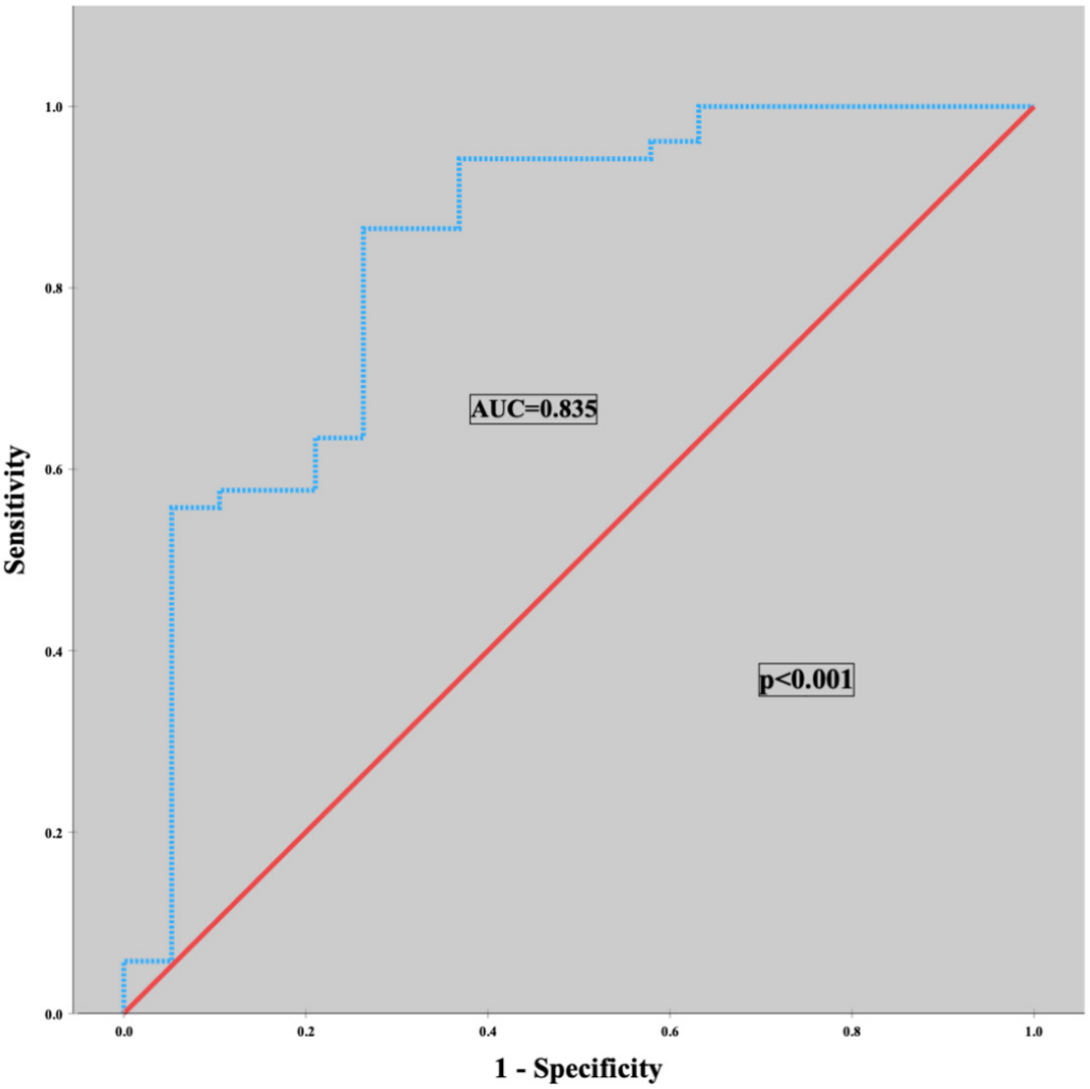
ROC analysis and chi-square test revealed a significant association between PNI and progression from BL to CR-POPF.

A significant association was detected between preoperative PNI value and progression from BL to CR-POPF in ROC curve analysis (AUC = 0.835; 95% CI, 0.717-0.953; *P* = .001). The optimum preoperative PNI cutoff value associated with progression was 37.4. This value was corrected to 38 for easy clinical use and validated with a chi-squared test (OR, 11.760; 95% CI, 3.430-40.322; *P* < .01); sensitivity and specificity were 73.7% and 80.8%, respectively (Figure 2). PPV and NPV of PNI were 58.3% and 89.4%, respectively. The rate of progression from BL to CR-POPF in patients with a preoperative PNI value ≥ 38 was 10.6% and with a PNI value < 38 was 58.3%.

Univariate and multivariate binary logistic regression analyses of the associations between clinicopathological characteristics and the progression from BL to CR-POPF are demonstrated in Table 3. The preoperative PNI value was the only independent risk factor (OR 15.428; 95% CI 3.714-64.085; *P* < .01).

## Discussion

In the present study, we found that the low preoperative PNI value is an independent predictive risk factor for progression from BL to CR-POPF after PD. Patients with 38 < PNI preoperatively, had a significantly higher progression rate. We think this result may create a different perspective about the early diagnosis and treatment of patients with BL who have high risk for progression to CR-POPF due to a low preoperative PNI value for pancreatic surgeons.

The hypercatabolism of cancer leads to malnutrition, which can cause negative postoperative surgical outcomes, especially in major surgeries.^12^ Pancreaticoduodenectomy is one of these most challenging operations and creates maximum metabolic stress with the inflammatory response that occurs with excessive tissue damage. Therefore, preoperative nutritional screening is extremely important. The PNI is one of these screening methods and is calculated by serum albumin level and total count of lymphocytes. In addition to immunonutrition, serum albumin and lymphocytes also play a role in tissue healing, collagen synthesis in the anastomosis, and inflammatory response.^13,14^ While there are studies showing the relationship between lower PNI values and negative postoperative results and poor prognosis in non-pancreatic cancers,^12,15^ studies about its negative effect on postoperative results of PD, especially including CR-POPF, are increasing recently.^13,16^ But as far as we know, this is the first study to present the PNI effect on the progression from BL to CR-POPF after PD.

Postoperative pancreatic fistula may develop due to leakage from the sutures passing through the pancreatic parenchyma or from minor ducts on the cut surface, ischemia in the remnant pancreas, or separation of the pancreaticojejunostomy anatomosis, although uncertain pathophysiologically.^7,17^ The first sign of POPF, as stated by ISPGS, is an increased amylase value of the drain fluid sample. Since this condition has no clinical relevance, it is called BL, and ‘wait and see’ approach begins for the pancreatic surgeon. BL, in addition to being a necessary and first step for diagnosis, is a ‘warning sign’ too. In our opinion, at this stage, decreased immunonutrition as a reflection of low preoperative PNI value prevents the limitation of inflammation triggered by BL and causes progression to the next stage, a CR-POPF. 

In our study, we detected the median progression time from BL to CR-POPF was 9 (5-19) days. In the current study by Raza et al., similar to our cohort, the time from BL to CR-POPF was approximately 9 (6-13) days postoperatively.^18^ As a result, it is not possible to diagnose a CR-POPF in an earlier postoperative period. Therefore, during this waiting period, planning accordingly such as keeping the intra-abdominal drain in place longer, early abdominal imaging, starting somatostatin analogs, and closely monitoring the postoperative follow-up may decrease the morbidity of progression to CR-POPF in high-risk patients, as similar to our BL group with a low preoperative PNI value.

Although routine intra-abdominal drain placement during PD is controversial, pancreatic surgeons mostly place, routinely.^8^ The main reason for this requirement is to diagnose POPF by examining the drain fluid on the 3rd postoperative day and to treat CR-POPF with the same drainage. It seems impossible to make this diagnosis early in patients without a drain. On the other hand, ‘when to remove the drain’ is a matter of greater debate. If these drains are not removed for a long time, they carry the risk of forming CR-POPF due to the erosion and inflammation they cause. Therefore, early removal of the drain reduces the risk of drain-related complications, especially CR-POPF.^19^ The ISGPS defined requiring a drain for more than 3 weeks after PD as Grade B POPF.^4^ However, no recommendation was made regarding which patients have BL, with low risk for progression to CR-POPF, could have the drain removed early, although this is the main concern of pancreatic surgeons. Even if there are no clinically relevant findings, it is inevitable for surgeons to want to keep the drain for a longer period as a precaution in patients with a high drain amylase value. As a result, we think that it is not possible to evaluate two patients whose drains were removed on the 3rd and 20th postoperative day in the same category of POPF. At this point, knowing the risk factors that will increase the progression from BL to CR-POPF, such as the preoperative low PNI value as we found in the present study, will make surgeons feel more comfortable and prevent the drain from remaining unnecessarily for a longer time. In fact, in our opinion, in order to draw clearer boundaries on this issue, patients with BL can be redefined into subgroups in the next update of the ISGPS, based on risk factors for progression to CR-POPF.

The preferred imaging method to detect intra-abdominal collections or hemorrhage, that develop due to CR-POPF after PD, is abdominal computed tomography (CT). Although postoperative CT is routinely performed in several institutions,^20^ the issue is controversial, especially in patients with BL. The reason for this is that the timing of intra-abdominal collections that may develop as the condition progresses from BL to CR-POPF is not clear. If the intra-abdominal drains placed perioperatively do not drain these collections, or are not in the correct location, new percutaneous or endoscopic drainages will be required. In the study by Cuellar et al,^21^ it was found that CT scans performed on the 7th postoperative day were insufficient to detect these collections and were false negative and it was stated that perhaps it would be more accurate to perform them on the 8th-10th days. As a result, we think that CT imaging in the period of 8-10 postoperative days in patients with a high risk for progression from BL to CR-POPF, such as low preoperative PNI value, may benefit pancreatic surgeons.

It is known that the anti-fistula effect of somatostatin analogs is theoretically by suppressing pancreatic exocrine secretions. While the routine use of somatostatin analogs to reduce POPF after PD is still controversial, there is no clear consensus on their use in the treatment of BL or CR-POPF. However, recent studies have shown that the use of these drugs is effective, especially in high-risk patients for CR-POPF.^22,23^ Based on the results of our study, in our opinion, prophylactic somatostatin analogs may benefit especially patients with BL and low preoperative PNI value, who have a high risk of progression to CR-POPF.

The timing to evaluate immunonutrition with biochemical markers is also still controversial in the literature. The main question is whether this evaluation gives more reliable results ‘before or after’ the operation. In their study, Kim et al^24^ found that these biochemical markers may change rapidly in the postoperative period and lose their reliability in assessing immunonutrition. The decrease of albumin levels results from various factors, such as capillary leakage into the interstitium as a result of increased vascular permeability, hemodilution, and reprioritization of hepatic protein synthesis from visceral proteins to acute phase reactant proteins.^25^ In parallel, we made PNI measurements preoperatively in our cohort, and we believe that this timing evaluates the nutritional status more realistically.

Therefore, the main question that comes to mind in the present study is, ‘Is nutritional support indicated, in patients with BL and have low preoperative PNI value, to prevent progression to CR-POPF?’. The ISPGS recommends nutritional support to reduce postoperative morbidity in patients undergoing pancreatic surgery, especially in the presence of hypoalbuminemia preoperatively.^26^ We hope that in the near future update, they may make a nutritional recommendation to prevent progression from BL to CR-POPF. Future studies on this subject may perhaps answer this question. We believe that providing nutritional support may help to keep patients stable in the BL stage and prevent progression to CR-POPF.

The strength of the present study is that the clinicodemographics and perioperative findings of the groups were homogeneous, except for the preoperative PNI value. A limited number of the patients and the retrospective design of the study are limitations.

In conclusion, the preoperative PNI value is an important parameter predicting the progression from BL to CR-POPF after PD. These high-risk patients with a preoperative PNI value < 38 and have BL may benefit from earlier diagnostic imaging, interventions, somatostatin analogs, longer drainage time, or nutritional support. More attention should be given to the possibility of patients having BL and low preoperative PNI value progressing to CR-POPF after PD.

## Availability of Data and Materials:

The data that support the findings of this study are available on request from the corresponding author.

## Figures and Tables

**Table 1.** Preoperative Findings and Definitive Pathological Results

Parameters	BL	CR-POPF	*P*
Age, years (mean ± SD)	62.8 ± 12.3	66.6 ± 9.9	.222
Gender (f), n (%)	21 (40.4%)	9 (47.4%)	.798
BMI (>30), n (%)	11 (21.2%)	3 (15.8%)	.745
HT (yes), n (%)	24 (46.2%)	12 (63.2%)	.317
COPD (yes), n (%)	3 (5.8%)	-	.559
CAD (yes), n (%)	3 (5.8%)	5 (26.3%)	.502
DM (yes), n (%)	16 (30.8%)	5 (26.3%)	.778
Smoking (yes), n (%)	9 (17.3%)	3 (15.8%)	1
Preoperative biliary drainage (yes), n (%)	25 (48.1%)	11 (57.9%)	.642
Previous abdominal surgery (yes), n (%)	12 (23.1%)	3 (15.8%)	.744
ASA score ≥3, n (%)	4 (7.7%)	4 (21.1%)	.197
Preoperative PNI, median (range)	41.6 (33.5-58)	35.6 (30.1-47.9)	**<.001**
Tumor location, pancreatic head, n (%)	15 (28.8%)	6 (31.6%)	1
Indication (malignant), n (%)	45 (86.5%)	17 (89.5%)	1

ASA, American Society of Anesthesiologists; BMI, body mass index; CAD, coronary artery disease; COPD, chronic obstructive pulmonary disease; DM, diabetes mellitus; HT, hypertension; PNI, prognostic nutritional index.

**Table 2.** Operative Findings and Postoperative Results

Parameters	BL	CR-POPF	*P*
Blood transfusion, unit median (range)	0 (0-6)	1 (0-4)	.053
Pancreatic texture, soft, n (%)	35 (67.3%)	15 (78.9%)	.511
Diameter of Wirsung, ≤3 mm, n (%)	25 (48.1%)	10 (52.6%)	.943
Operation time, minutes, median (range)	420 (240-780)	360 (240-720)	.686
Postoperative CRP, mean ± SD	179.8 ± 67.4	209 ± 83.2	.122
Wound infection (yes), n (%)	15 (28.8%)	3 (15.8%)	.362
DGE, Grade B+C (yes), n (%)	9 (17.3%)	3 (15.8%)	1
CD ≥3 complications, n (%)	6 (11.5%)	11 (57.9%)	**<.001**
Re-operation (yes), n (%)	2 (3.8%)	4 (21.1%)	**.04**
Length of hospital stay, median (range)	13 (6-30)	21 (8-57)	**<.001**
Mortality in 90 days, n (%)	-	3 (15.8%)	**.017**

CD, Clavien–Dindo; CRP, C-reactive protein; DGE, delayed gastric emptying.

Statistically significant values are in bold.

**Table 3.** Uni- and Multivariate Logistic Regression Analysis of Predictors for Progression From BL to CR-POPF

Variable	Univariate Analysis		Multivariate Analysis	
OR (95% CI)	*P *	OR (95% CI)	*P *
Age	1.031 (0.982-1.082)	.221	1.007 (0.946-1.072)	.821
ASA score <3				---
ASA score ≥3	3.200 (0.712-14.374)	.129	6.341 (0.789-50.955)	.082
Postoperative CRP	1.006 (0.988-1.014)	.126	1.010 (1.000-1.019)	.050
Intraoperative blood transfusion	1.312 (0.861-2.000)	.207	1.258 (0.733-2.157)	.405
PNI ≥38				---
PNI <38	11.760 (3.430-40.322)	<.01	15.428 (3.714-64.085)	<.01

ASA, American Society of Anesthesiologists; CR-POPF, clinically relevant postoperative pancreatic fistula; OR, odds ratio; PNI, prognostic nutritional index.
